# Effect of Active and Assisted Living technologies on psychosocial well-being in older adults: systematic review

**DOI:** 10.3389/fpubh.2025.1717154

**Published:** 2026-01-08

**Authors:** Carlo Giacomo Leo, Antonella Bodini, Maria Rosaria Tumolo, Saverio Sabina, Riccardo Colella, Andrea Brancaccio, Virginia Recchia, Pierpaolo Mincarone

**Affiliations:** 1 Institute of Clinical Physiology, National Research Council, Lecce, Italy; 2 MOVE-mentis s.r.l., Cesena, Italy; 3 Institute for Applied Mathematics and Information Technologies “E. Magenes”, National Research Council, Milan, Italy; 4 Institute for Research on Population and Social Policies, National Research Council, Brindisi, Italy; 5 Department of Innovation Engineering, Faculty of Engineering, University of Salento, Lecce, Italy

**Keywords:** Active and Assisted Living (AAL), health-related quality of life (HRQoL), older adults, psychosocial outcomes, psychosocial well-being

## Abstract

**Background:**

In a rapidly aging society, the concept of psychosocial well-being becomes increasingly relevant, independent of health. Indeed, psychosocial well-being is closely related to autonomy, independence, and self-efficacy. Technological approaches that support older adults in leading active, healthy, and independent lives are framed within the concept of Active and Assisted Living (AAL). However, evidence regarding their impact on psychosocial well-being remains limited.

**Objective:**

This systematic review evaluates the psychosocial effects of AAL technologies in older adults.

**Methods:**

We included intervention studies reporting psychosocial outcomes related to older adults’ use of AAL technologies. We excluded studies involving participants receiving continuous on-site healthcare assistance or with moderate to severe mental health problems, technologies restricted to basic home automation or lacking advanced data processing and automation, and interventions focused on rehabilitation after acute events. Studies using not-validated measures or without quantitative evaluation of outcomes were also excluded. Searches were conducted in MEDLINE, HealthSTAR, Cochrane Central Register of Controlled Trials, IEEE Xplore, APA PsycArticles, Scopus, and Web of Science from inception to January 2025. Key attributes of eligible publications were critically discussed using a 2 × 2 logic which frames findings by both technological performance and psychosocial outcome.

**Results:**

Of 6,349 records identified, 15 independent studies were included. AAL solutions targeted promotion or guidance of physical activity, self-management of chronic conditions, fostering healthy and safe habits or distraction from pain. Twelve studies assessed health-related quality of life (HRQoL) and close concepts. Depression was the second most investigated outcome, followed by fear of falling, with QoL and loneliness also reported.

**Conclusion:**

This review offers a critical analysis of both the findings and the methodologies employed in the selected studies, acknowledging the complexity of drawing unequivocally positive or negative conclusions regarding the impact of AAL technologies on the psychosocial well-being of older adults. The results highlight the need for a shared conceptual framework to inform the design, assessment, and validation of technologies intended to support daily living.

## 1 Introduction

While physical health is generally expected to decline with age, other aspects of a fulfilling life, those falling under the broader definition of well-being (1), become increasingly prominent. It is well established, in fact, that, particularly among older adults, well-being is inherently multidimensional also encompassing emotional, psychological, social, developmental, and financial domains (2). Within this framework, emotional, psychological and social domains, which collectively constitute the concept of psychosocial well-being (3), are closely associated with constructs such as autonomy, independence, and self-efficacy (4). As people age, maintaining well-being calls for a broader rethinking of how longer lives are approached and supported. This perspective is captured in the growing call for a transition from an aging society to a longevity society, which redefines extended lifespans as an opportunity to redesign the life course (5). Technological, social, and organizational approaches that support older individuals in leading active, healthy, and independent lives are framed within the concept of *Active and Assisted Living* (AAL) (6). Originally, the acronym AAL referred to *Ambient Assisted Living* and referred to technologies focused on safety measures, such as fall detection and emergency recognition (7), basic home automation tools (e.g., lighting, climate control, or access systems) or standard communication applications (e.g., videoconferencing or messaging platforms). These technologies lack in analytic or adaptive capabilities. Nowadays, driven by the cultural shift toward a longevity society, the integration of wearable devices, innovative gaming technologies, and more intuitive user interfaces capable of generating real-time feedback, adaptive support, or automated interpretations has progressively expanded the scope of AAL, thus allowing the transition from “*Ambient*” to “*Active*” *Assisted Living* (8). This concept encompasses self-monitoring, self-management, and the continuous collection of longitudinal data (9) with the primary objectives to promote self-empowerment, personalize healthcare, and support daily activities by accommodating evolving individual capabilities. Ultimately, these strategies enable older adults to maintain a more independent and self-determined lifestyle in their home and community (10) for as long as possible (7, 11). Consequently, AAL technologies should be evaluated based on their capacity to support the multidimensional needs of aging individuals. Although numerous AAL initiatives have been promoted and funded also by EU, their impact on the psychosocial well-being of older adults – and the roles these technologies play within the complex and multifaceted contexts of aging – remain largely unknown (12).

Systematic reviews have highlighted the potential of AAL technologies to improve health outcomes in older adults, including changes in physiological parameters, symptoms, and clinical indicators (13–18). However, the impact on psychosocial well-being of aging individuals remains insufficiently explored. Our systematic review aims to address this gap by evaluating the effects of AAL solutions on a range of psychosocial outcomes.

## 2 Methods

The PRISMA (Preferred Reporting Items for Systematic Reviews and Meta-Analyses) guidelines (19–21) were followed. A protocol was prepared only for internal purposes.

### 2.1 Eligibility criteria

The eligibility criteria are listed in Table 1.

**Table 1:** Eligibility criteria.

Parameter	Inclusion criteria	Exclusion criteria
Population	Older people: study population with a mean or median age of 60 years and more	Subjects receiving continuous on-site healthcare assistance. Individuals with moderate and severe mental health problems.
Intervention	Active and Assisted Living solutions	Technologies limited to home automation applications. Communication tools that do not inherently include or are not part of systems with advanced data processing and automation features. Technologies for rehabilitation for acute events.
Comparator		No restriction
Outcome	Psychosocial well-being	Outcomes assessed through not-validated measures. Lack of a quantitative evaluation of the technology’s effect on the outcome of interest.
Study type	Interventional study	Qualitative methodology. Application of AAL technology not distinct from other types of intervention.
Publication type		Literature reviews, grey literature, conference proceedings and unpublished material.
Language		No restriction
Publication period		No restriction

A common convention defines individuals aged 65 years and older as older adults (22) but lower age thresholds are not uncommon (23). Consequently, we implemented a nuanced criterion to ensure a comprehensive representation of older adults, including studies in which the mean or median age of participants was ≥60 years. This approach accommodates cultural variability in the definition of older age while maintaining a clear focus on the target population.

Our search was not restricted by the presence of a comparator, language, geographic context, funding source or time horizon.

We excluded studies on general populations that did not provide distinct findings pertaining to older adults and works in which subjects received continuous on-site healthcare assistance, as this review focuses on independent living. Moreover, we excluded studies involving subjects with mental impairments significantly interfering with or restricting one or more major life activities and whose substantial cognitive decline significantly hampers both social and occupational functioning.

We did not consider home automation applications (such as home climate control, access management, and lighting management) and communication tools (e.g., text messaging applications or video conferencing between a physician and a patient) that do not inherently include, or are not part of, systems featuring advanced data processing algorithms for real-time data analysis and interpretation. Interventions designed solely for short-term rehabilitation were also excluded, as not directly applied to maintaining independence in activities of daily living. Other pathologies, however serious, were not excluded, precisely to be able to verify whether certain clinical conditions (e.g., COPD/PD) are more or less amenable to psychosocial impact via AAL.

We relied on outcomes measured through validated self-reported questionnaires that assess general psychological or social functioning, instruments used for clinical screening of symptoms, and measures of perceptions related to everyday activities. Any reference to a specific construct reflects the terminology adopted by the original authors of the study or, when not explicitly stated, by relevant literature. These designations do not represent any theoretical attribution on our part. Self-reports developed solely for monitoring treatment adherence or behavior tracking were excluded, as they do not directly capture psychosocial outcomes. Including objective cognitive performance measures would conflate cognitive capacity with psychosocial experience and undermine the clarity of the analysis.

We excluded studies using qualitative research methods (such as interviews, focus groups, etc.), observational studies, and those lacking a validated method for measuring the outcome of interest or a quantitative evaluation of the technology’s effect.

Literature reviews, grey literature, conference proceedings, and unpublished material were not considered.

### 2.2 Information sources

We searched the following electronic databases up to January 2025: MEDLINE(R) and Epub Ahead of Print, In-Process, In-Data-Review & Other Non-Indexed Citations, Daily and Versions(R), Healthstar, Cochrane Central Register of Controlled Trials, through the OVID platform, IEEE Xplore, APA PsychArticles, Scopus and Web of Science. Additionally, using a “snowballing” approach (24), we manually screened the reference list of included papers and conducted a systematic citation tracking in Scopus, PubMed and Google Scholar.

### 2.3 Search strategy

We developed search strings using the PICO framework to structure the research question. We selected search terms to identify older adults, AAL technologies, psychosocial well-being, and interventional trials. To enhance sensitivity, we used the MeSH terms, entry terms, and synonymous. The complete search strategy is provided as Supplementary file 1.

Duplicates were removed using an automatic procedure based on PubMed IDs and DOIs in Microsoft Excel, supplemented by visual inspections of studies with identical titles.

### 2.4 Selection process

Search results were retrieved from the databases and independently double-screened by all the authors. The initial screening, supported by the Rayyan tool (25), was based on the title and abstract. A pilot phase involving 100 studies was conducted to refine and clarify the eligibility criteria, ensure consistency among reviewers, and train the research team. Subsequently, potentially relevant papers were retrieved for full-text screening, and their eligibility was determined as previously described. Rayyan was adopted also for full-text assessment. Any disagreements were resolved through plenary discussion among all the authors until consensus was reached.

### 2.5 Data collection process

Each selected study was randomly assigned to two authors who independently extracted relevant data. Discrepancies were resolved as described above. Each study was assessed for methodological issues by a statistician and a psychometrician.

### 2.6 Data items

We extracted three different types of information. First, we recorded the general study characteristics such as context (country, time period and setting), population (sample size, age, health condition), study design and implementation setting. Second, we described the intervention, with details on the intended effect, type and functionalities, supervision or assistance provided in the use of AAL technology, and non-technological components, and, where applicable, the comparator. The AAL technologies were further characterized with respect to their technological components. Finally, we considered the effect on psychosocial well-being as reported in Section 2.1.

### 2.7 Synthesis of evidence

Data were presented in tabular form. A narrative synthesis was then conducted to provide an overall summary of the findings from the selected studies, with a detailed discussion of their biases, strengths, and limitations.

## 3 Results

**Figure 1:**
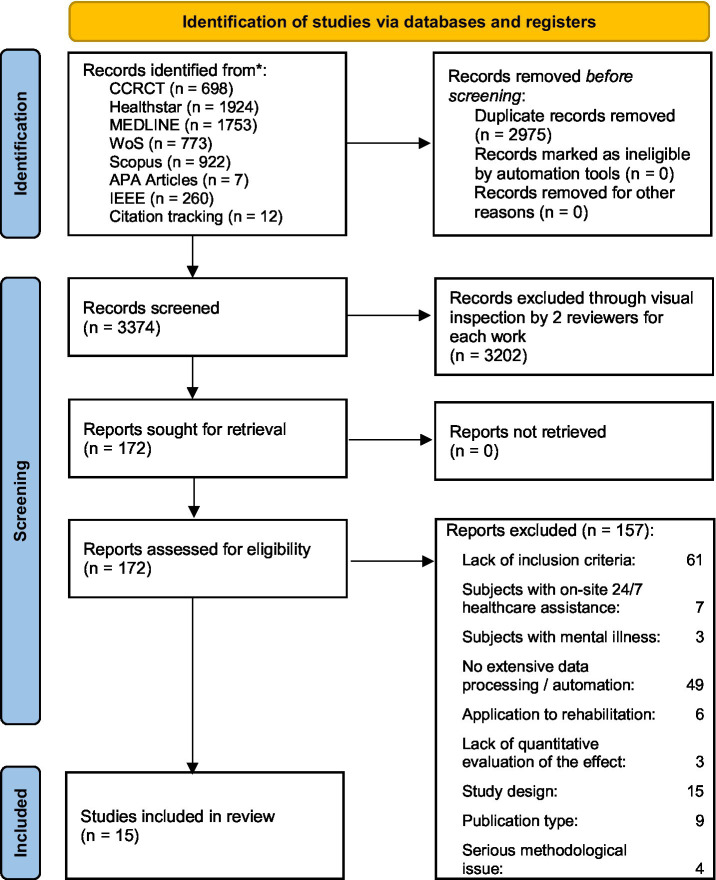
PRISMA flow diagram.

A total of 6,349 records were identified of which 15 independent studies were included in this review (Figure 1; Table 2). During the title–abstract stage, many records were excluded because they clearly fell outside the scope of the review (e.g., non-target populations, interventions unrelated to AAL, or non-primary publication types). Detailed reasons for exclusion at the full-text stage are provided in the Supplementary Table 1, where each of the 157 excluded studies is associated with the specific criterion applied.

**Table 2:** Key characteristics of included studies.

Study	General information			Methodology					Participants	
	Initiation	Country	Aim	Design and extent	Intervention Schedule	Study setting	Sample Size	Age eligibility	Sample Age (mean, SD)	Females %
Benham S et al., 2019 (26)	NR	United States	To examine the applicability and effectiveness of an immersive virtual reality intervention for pain, depression, and QoL in older patients experiencing pain	Exploratory Pre-post	6 weeks 2 sessions/weeks 15–45 min/session	Senior day center	IG: 12	Yes (≥55)	IG: 70.2 (3.6)	IG: 87.5%
Boer L et al., 2019 (36)	2015	Netherlands	To examine the effects (in terms of weeks without exacerbations, health status, self-efficacy and self-management behaviors, health care utilization) of a mHealth tool that supports patients with COPD in the self-management of exacerbations compared with the use of a paper exacerbation action plan	Multicenter RCT	52 weeks	Home	IG: 43 CG: 44	No	IG: 69.3 (8.8) CG: 65.9 (8.9)	IG: 42% CG: 34%
Chan RSM et al., 2024 (32)	2022	China (Hong Kong)	To evaluate the efficacy in improving oral functions and user experience of healthy adults of a mobile application for facial movement tracking during oral muscle training program	Multicenter Pre-post	8 weeks up to daily	Home	IG: 70	No	IG: 67.70 (4.93)	IG: 74.3%
Dieter V et al., 2024 (35)	2020	Germany	To evaluate the efficacy of a fully automated app-based exercise intervention, with and without a supporting knee brace, on health-related outcomes, performance measures, and adherence in patients with knee osteoarthritis, compared to not receiving any study intervention or instruction for changing normal habits	3 arms Pilot RCT	12 weeks 3 sessions/week 30 min/session	Home	IG1: 15 IG2: 15^A^ CG: 31	No	IG1 + IG2: 61.5 (7.5) CG: 64.2 (9.3)	IG1 + IG2: 40% CG: 58%
Ginis P et al., 2016 (40)	NR	Belgium and Israel	To test the feasibility of gait training with the CuPiD-system in the home environment. To discover the differential effects of CuPiD training versus conventional home-based gait intervention on gait, balance and HRQoL in patients with PD.	Multicenter Pilot RCT	6 weeks 3 sessions/week 30 min/session (for subjects without FOG) 60 min/session (for subjects with FOG) + 4 weeks for follow-up	Home	IG: 22 CG: 18	No	IG: 67.30 (8.13) CG: 66.11 (8.07)	IG: 15% CG: 27.8%
Gschwind Y et al., 2015 (27)	2014	Germany, Spain and Australia	To investigate the feasibility and effectiveness of the individually tailored iStoppFalls exergame program on fall risk factors in older adults, compared to a control group that received no additional intervention and was encouraged to follow their habitual exercise routines if applicable	Multicenter RCT	16 weeks 120 min/week (for balance exergames) and 60 min/week (for strength exercises) + 8 weeks for follow-up	Home	IG: 78 CG: 75	Yes (≥ 65)	IG: 74.7 (6.7) CG: 74.7 (6.0)	IG: 55.8% CG: 66.7%
Keogh A et al., 2024 (38)	2021	Ireland	To examine the usability, adherence, and feasibility of a digital health tool to support effective self-care behaviors in patients with HF. To assess the impact of the tool on self-care behaviors and QoL.	Pilot Pre-post	26 weeks	Home	IG: 17	No	IG: 72 (9.9)	IG: 35%
Lee M et al., 2015 (34)	NR	Republic of Korea	To determine if individualized feedback-based virtual reality exercise is effective in improving HRQoL in older women compared to a group-based exercise group	RCT	8 week 3 sessions/week 60 min/session	Exercise rooms within university facilities	IG: 26 CG: 28	No	IG: 68.77 (4.62) CG: 67.71 (4.31)	IG: 100% CG: 100%
Liljeroos M et al., 2024 (39)	2019	Sweden	To evaluate the effects of using an mHealth tool on self-care behavior and to explore the experiences and perceptions of patients with HF about the tool	Multicenter Pre-post	52 weeks	Home	71	No	IG: 76.7 (6.6)	IG: 49%
Lim JS et al., 2023 (28)	2021	Republic of Korea	To investigate whether a social robot intervention is effective in improving cognitive function, depression, loneliness, and quality of life in older adults living alone, compared to a control group that did not participate in the program	non-randomized controlled trial	6 weeks 2 sessions/week 50 min/session	Senior day center	IG: 36 CG: 36	Yes (≥65)	IG: *75.4* CG: *78.4*	IG: 93.5% CG: 84.8%
Park YH et al., 2021 (33)	2017^B^	Republic of Korea	To examine, in patients with prostate cancer on androgen deprivation therapy, whether the Smart After-Care service, a lifestyle intervention, affects clinical outcomes compared to face-to-face education and a paper brochure with similar contents	multicenter RCT	12 weeks	Home	IG: 86 CG: 86	No	IG: 66.3 (6.8) CG: 66.5 (8.2)	IG: 0% CG: 0%
Radhakrishnan K et al., 2021 (29)	2019	United States	To compare daily weight monitoring and physical activity behavior adherence by older patients diagnosed with HF using a sensor-controlled digital game intervention versus a sensors-only intervention.	Pilot RCT	12 weeks + 12 weeks for follow-up	Home	IG: 19 CG: 19	Yes (≥55)	IG1: *67.9* CG: *64.7*	IG1: 47% CG: 47%
Seok JW et al., 2022 (30)	2019	Republic of Korea	To examine the feasibility of Touch-Care system, which combined life logging (i.e., tracking user’s daily life, monitoring health-related behaviors, motivating) with human body communication technology, and its effect on the physical and psychological status of older adults living alone.	Pilot Pre-post	22 weeks	Home	IG: 22	Yes (≥65)	IG: 77.3 (3.8)	IG: 78.6%
Wu R et al., 2024 (37)	2019	Canada	To determine if patients with COPD will use a self-management application for smartphones and smartwatches and to determine the effects on their self-management.	Pilot Pre-post	26 weeks	Home	IG: 34	No	IG: 69.8 (9.1)	IG: 50%
Yang WC et al., 2016 (31)	2011	Taiwan	To test if the home-based virtual reality balance training is more effective than the conventional home balance training in improving balance, walking, and quality of life in patients with PD	RCT	6 weeks 2 sessions/week 50 min/session + 2 weeks for follow-up	Home	IG: 11 CG: 12	Yes [55–85]	IG: 72.5 (8.4) CG: 75.4 (6.3)	IG: 36.4% CG: 41.7%

Intervention Schedule reports duration, frequency, session length and follow-up. Italics indicate data derived by the authors. ^A^In IG2 the mHealth intervention was associated to a non-technological intervention and we have not considered this group in our analyses. ^B^Based on data reported in ClinicalTrials.gov. CG, Control group; COPD, Chronic obstructive pulmonary disease; FOG, Freezing of gait; HF, Heart failure; IG, Intervention group; mHealth, mobile health; NA, Not applicable; NR, Not reported; QoL, Quality of life; PD Parkinson’s disease; RCT, Randomized controlled trial.

### 3.1 General characteristics of the study populations

Most studies were conducted in Asia (40%, 6/15) and in Europe (40%, 6/15); the others were conducted in America. Less than half of the studies explicitly used an age-based criterion (26–31). In other cases, the inclusion of older adults was incidental, either a consequence of recruiting strategies (32) or because the condition under investigation is more prevalent in older populations. In studies involving all genders, the percentage of women ranged from 35 to 93.5%. One study was specific for men due to its clinical emphasis on prostate cancer (33), while another considered only women, although with no justification (34).

All but five studies (27, 28, 30, 32, 34) addressed a specific health condition (pain (26), osteoarthritis (35), chronic obstructive pulmonary disease (COPD) (36, 37), heart failure (HF) (29, 38, 39), Parkinson’s disease (PD) (31, 40), or prostate cancer (33)).

### 3.2 Study design and conduction

Nearly half of the included studies are reported as feasibility or pilot trials by the study authors, reflecting a research landscape still in an exploratory phase. Eight studies (27–29, 31–33, 35, 37) reported sample size determination. All but one (37) relied on traditional power analysis methods associated with a specific variable (27, 29, 31) or on general Cohen effect size considerations, but without specifying the reference variable. The mean intervention duration was 18 weeks (range: 6–52). Only four studies (27, 29, 31, 40) included follow-up assessments (2–12 weeks). All studies were conducted in participants’ homes, except three: two in non-residential day centers for older adults (26, 28), and one (34) in university-based exercise rooms.

Eight studies (27, 29, 31, 33–36, 40) were randomized controlled trials, one study was a non-equivalent control group pre-test–post-test design (28) and the other six studies adopted a single-arm pre-post design. Of the controlled studies, four (27, 28, 35, 36) employed comparators receiving no interventions beyond educational advice or the recommendation to continue with usual care. The other five include comparators subjected to treatments (29, 31, 33, 34, 40).

### 3.3 Interventions: objectives and technological implementation

The AAL technologies proposed in the selected studies (Table 3) were designed to promote or guide physical activity (27, 31, 32, 34, 35, 40), support self-management of medical conditions (29, 36–39) or address both domains simultaneously (33). Other interventions aimed to foster healthy and safe habits (30) or to distract from pain (26). The majority of the proposed technologies consist of mHealth (i.e., mobile health) app-based systems that offer feedback on performance and encourage self-monitoring of routines and related outcomes, or virtual reality platforms that deliver instructions and real-time feedback on task execution. A notable exception is the social robot PIO designed for cognition-enhancing training, gymnastic exercises, and emotional support (28).

**Table 3:** Interventions and comparators.

Study	Intervention				Comparator
	Objective	Type and Functionality	Supervision or assistance provided in the use of AAL technology	Co-intervention	
Chan RSM et al., 2024 (32)	Guiding and controlling physical exercise program	AI-enabled mHealth app-based system (*e-Oral*) adopting device sensors for continuous data collection. The application provides visual-audio demonstrations, feedback and performance tracking in oral muscle training program.	Supervision and safety monitoring: Regular video/phone consultations with Speech therapists every two weeks. Technical support: Regular video/phone consultations with Speech therapists every two weeks.		NA
Dieter V et al., 2024 (35)	Guiding and controlling physical exercise program	Non-Immersive mHealth VR Motion-Tracking System (*Re.flex* by Kineto Tech Rehab SRL) to guide the physical exercise	Supervision and safety monitoring: Medical assistance through app messenger service. Technical support: Not reported.		Usual care (i.e., any prescribed pharmacological or physical interventions typically indicated for knee osteoarthritis)
Ginis P et al., 2016 (40)	Guiding and controlling physical exercise program (gait training)	mHealth app-based system (*CuPiD-system*) with connected non-medical devices for continuous data collection. The system provides auditory feedback on how to (a) walk according to ACSM’s exercise guidelines (57); (b) avoid FOG (only for the participants with FOG).	Supervision and safety monitoring: weekly home visits. Technical support: phone assistance if needed.		Received practice schedule, recommendations for training and weekly assistance as the IG
Gschwind Y et al., 2015 (27)	Guiding and controlling physical exercise program	Non-Immersive VR Motion-Tracking System (*iStoppFalls*) to conduct exergames.	Supervision and safety monitoring: Two weeks after system installation, home visit to ensure correct and safe system use as well as progression of training; additional home visits if required. Technical support: Phone support	Evidence-based educational booklet about general health and fall	Habitual exercise routines, if applicable. Same co-intervention as for IG
Lee M et al., 2015 (34)	Guiding and controlling physical exercise program	Non-Immersive VR Motion-Tracking to guide physical exercise	Supervision and safety monitoring: A research assistant was present to monitor participants for emergencies. Technical support: On-site		Physiotherapist guided group-based exercise program adapted from Donat and Ozcan, 2007 (58), similar to *Zen Energy*.
Yang WC et al., 2016 (31)	Self-management of physical activity (balance training)	Non-Immersive VR center-of-pressure Motion-Tracking System for exergaming	Supervision and safety monitoring: A home physiotherapist guided warm-up stretching, ensured appropriate execution and supervised for safety. Technical support: Not reported.		Conventional balance training (as detailed in the original study), delivered in-person by a home physiotherapist who provided manual guidance and verbal instruction
Park YH et al., 2021 (33)	Guiding physical exercise program, allowing daily activity and diet monitoring, allowing monitoring of health parameters (blood pressure and glucose level), providing counseling by physicians, clinical nutritionists, and exercise therapists, and delivering health information.	Hybrid mHealth app-based and web-based system (*Smart After-Care*) with a diary and connected medical and non-medical devices for discrete and continuous data collection. The system allows for (1) individually prescribed exercise programs; (2) physical activity tracking; (3) diet tracking and monitoring; (4) comorbidity management including blood pressure and glucose levels; (5) counseling by physicians, clinical nutritionists, and exercise therapists; and (6) provision of health information	Supervision and safety monitoring: Not reported. Technical support: Not reported.		Face-to-face education, a paper brochure covering the contents of the IG program, and a conventional activity tracker used to record daily step count, minutes of physical activity, and the number of weekly resistance exercise sessions.
Boer L et al., 2019 (36)	Self-management of medical condition	mHealth app-based system (*ACCESS*) with questionnaire and connected medical devices for discrete data collection. The system employs algorithms for treatment recommendations	Supervision and safety monitoring: Not reported Technical support: During 2 training weeks	Educational session based on the Dutch version of the *Living Well with COPD* self-management program (59, 60)	Exacerbation self-management support using a paper exacerbation action plan (*Living Well with COPD* (59, 60) when not already possessed by the subject). Same co-intervention as for IG
Keogh A et al., 2024 (38)	Self-management of medical condition and self-care	mHealth app-based system with a questionnaire and connected medical and non-medical devices for discrete and continuous data collection. The application supports (1) self-care maintenance (e.g., taking or adjusting medication, engaging in physical activity, and adhering to a healthy diet), (2) self-care monitoring (e.g., regular weighing), and (3) self-care management (e.g., changing treatment dose in response to symptoms)	Supervision and safety monitoring: Not reported. Technical support: Not reported.		NA
Liljeroos M et al., 2024 (39)	Self-management of medical condition	mHealth app-based system (*OPTILOGG*) with a questionnaire and connected devices for discrete data collection. The system employs algorithms for treatment recommendations and interactive health education	Supervision and safety monitoring: Patients were scheduled for regular appointments at the HF clinic. Technical support: Not reported.		NA
Radhakrishnan K et al., 2021 (29)	Self-management of medical condition	Sensor-Controlled Digital Game mobile app (data from sensors trigger game progress, rewards, personalized and contextually relevant feedback, and incentives based on participants’ real-time behaviors)	Supervision and safety monitoring: Phone assistance in case of poor data (only 1st week). Technical support: Phone assistance.	Smartphone App (*Health Mate*, Withings, France) for monitoring fitness, activity, and health (connected with an activity tracker and weighing scale)	Same co-intervention as for IG
Wu R et al., 2024 (37)	Self-management of medical condition	mHealth app-based system with a diary and connected medical and non-medical devices for discrete and continuous data collection. The application provides functionality to monitor and display health data and activity. Feedback and motivational messages are provided for self-management. Other features include educational COPD resources, video exercises, medication reminders.	Supervision and safety monitoring: Little reinforcement for ongoing use provided. More robust regular interactions recommended as known to increase patient activation (health coaching and motivational interviewing [19], monthly calls). Technical support: Not reported.		NA
Seok JW et al., 2022 (30)	Promoting healthy and safe habits	AI-Driven Digital Health System (*TouchCare*, DNX Co. Ltd., Republic of Korea) with connected non-medical devices and touchtags. The system adopts life logging (it registers touched selected items and measures physical activity) and provides voice messaging to trigger user’s positive behavioral change, decrease loneliness, and promote safe activity in order to minimize accidents	Supervision and safety monitoring: Not reported. Technical support: Not reported.		NA
Benham S et al., 2019 (26)	Distracting from pain	Immersive VR System (*Vive*, HTC Corp, Taiwan) for gaming – featuring engagement with pets, exploration of animals, interactive music, and travel	Supervision and safety monitoring: A researcher through a computer monitor display. In each session, participants were given instructions for safety and were requested to declare any adverse symptom Technical support: On-site		NA
Lim JS et al., 2023 (28)	Allowing cognition-enhancing training, providing emotional support and conducting gymnastic exercises	Parrot shaped social robot (*PIO*, Why Dots Inc., Republic of Korea) that integrates a computer-assisted cognitive and emotional rehabilitation program and teaches movements for gymnastics purposes. The social robot was used in group sessions.	Supervision and safety monitoring: For smooth progress, two research assistants were used. Technical support: On-site	Usual care (music, art, programs, etc.)	Usual care

Studies are ordered based on their objective. Co-intervention refers to any additional care, treatment, or support provided alongside the primary intervention or comparator, which may influence study outcomes. Training/support on how the system worked is not reported. COPD, Chronic obstructive pulmonary disease; FOG, Freezing of gait; HF, Heart failure; IG, Intervention group; mHealth, mobile health; NA, Not applicable; VR, Virtual reality.

All the technologies included in this review, except for four studies (30, 33, 36, 38), explicitly stated the need for supervision and safety monitoring by researchers or healthcare personnel. It is important to note that, in some cases, non-AAL components were used as co-interventions, such as the provision of educational material (27, 36), music and art programs (28), or a supplementary basic smartphone app for fitness and weight tracking not meeting our criteria for AAL technologies (29).

The technological components of the AAL solutions are detailed in Supplementary Table 2.

### 3.4 Psychosocial outcomes and related measurement tools

For the terminology adopted in this section, we refer to Section 2.1 and Table 4.

**Table 4:** Summary of study findings.

Study	Design	Technology	Effects on technology related outcomes^A^	Effects on psychosocial wellbeing			
				Outcome^B^	Measurement tool	Efficacy	
						Time effect	Group effect
Benham S et al., 2019 (26)	Pre-post	Immersive VR system for distracting from pain, depression, and QOL in older patients experiencing pain	Yes (pain reduced)	QoL	WHO Quality-of-Life Scale (WHOQOL-BREF)	No SS change	
				Emotional Distress–Depression	PROMIS® Item Bank v. 1.0– Emotional Distress–Depression	No SS change	
Boer L et al., 2019 (36)	RCT	mHealth app-based system for Self-management of exacerbation versus paper action plan in patients with COPD	No SS difference between groups (change of self-reported self-management behavior)	Health status	Nijmegen Clinical Screening Instrument (NCSI) – (Physiological functioning domain excluded by authors)	No SS change	No SS difference
				Health status	Clinical Chronic Obstructive Pulmonary Disease Questionnaire (CCQ)	No SS change	No SS difference
				HRQoL	EuroQol-5D (EQ-5D) (with VAS)	No SS change	No SS difference
Chan RSM et al., 2024 (32)	Pre-post	AI-enabled mHealth app-based system for guiding and controlling physical exercise program in adults (oral muscle training program)	Yes (oral motor functions post-training and in the maintenance phase; heavier mouth dryness after training)	HRQoL	Oral Health Impact Profile-14, Chinese version	No SS change post-training and at maintenance	
Dieter V et al., 2024 (35)	RCT	Non-Immersive mHealth VR Motion-Tracking System for guiding and controlling exercise therapy versus a waiting list in patients with knee osteoarthritis	*No analysis performed in groups of interest*	HRQoL	Knee Injury and Osteoarthritis Outcome Score (KOOS)		Statistically significant superiority of IG versus CG for pain, physical function, and QoL, adjusted for baseline.
Ginis P et al., 2016 (40)	RCT	mHealth app-based system for guiding and controlling physical exercise versus conventional home-based gait exercise program in patients with PD	Yes (better balance in IG at post-test but not at follow-up).	HRQoL	Short-Form Health Survey (SF-36)	No SS change in both PCS and MCS *The 8 subscales were not analyzed*	Significant time × group interaction for PCS: the CG experienced a decrease in self-reported physical health at follow-up, while the IG group did not. *The 8 subscales were not analyzed*
				Fall fear	Falls Efficacy Scale-International (FES-I)	No SS change	No SS difference
Gschwind Y et al., 2015 (27)	RCT	Non-Immersive VR Motion-Tracking System for guiding and controlling physical exercise to prevent falls versus habitual exercise routines in older adults	Yes (lower fall risk and better dual-task walking test time in IG; worse hand reaction time in CG)	HRQoL	EuroQol-5D (EQ-5D) (with VAS)	No SS changes	Not difference
				HRQoL	12-item WHO Disability Assessment Schedule (WHODAS)	No SS change	No SS difference
				Depression	9-item Patient Health Questionnaire (PHQ-9)^C^	No SS change	No SS difference
				Fall fear	Shortened Iconographical Falls Efficacy Scale (Icon-FES)	No SS change	No SS difference
Keogh A et al., 2024 (38)	Pre-post	mHealth app-based system for self-care behaviors and quality of life in patients with HF	No SS changes (self-care)	HRQoL	Minnesota Living with HF Questionnaire (MLwHFQ)	No SS change	
Lee M et al., 2015 (34)	RCT	Non-Immersive VR Motion-Tracking system for guiding and controlling physical exercise to improve HRQoL versus group-based exercise in older women	Yes (higher leg strength and endurance in IG; better aerobic endurance and dynamic balance in both groups)	HRQoL	36-item Short-Form Health Survey (SF-36)	Significant improvement in role-physical, bodily pain, general health, vitality, role emotional, mental health and MCS in IG Significant improvement in role physical, general health, social functioning and PCS in CG	Significant difference between IG and CG in MCS, with greater improvement in IG than CG
Liljeroos M et al., 2024 (39)	Pre-post	mHealth app-based system for self-care behavior of patients with HF	Yes (improved self-care behavior at 3 but not at 12 months)	Depression	Hospital Anxiety and Depression Scale (HADS)	No SS change from baseline to 3 months and again at 1 year follow-up	
				Perceived control	Control Attitudes Scale (CAS)	No SS change from baseline to 3 months and again at 1 year follow-up	
Lim JS et al., 2023 (28)	nRCT	Social robot for cognition-enhancing and emotional support versus usual habits in older adults	Yes (improved cognitive function in IG; worsened in CG)	Depression	Geriatric Depression Scale Short Form (GDS), Korean version	Note: The significance of pre-post change was not analyzed in the study	Significant difference between IG and CG with greater improvement in IG than CG^D^
				Loneliness	Revised UCLA Loneliness Scale (RULS), Korean version	Note: The significance of pre-post change was not analyzed in the study. *Significant within group variation in IG only*	Significant difference between IG and CG with greater improvement in IG than CG
				HRQoL	EuroQol-5D (EQ-5D) (*without* VAS)	Note: The significance of pre-post change was not analyzed in the study	Significant difference between IG and CG in the mobility subscale, with greater improvement in IG
Park YH et al., 2021 (33)	RCT	Paired hybrid mHealth app-based and web-based system for lifestyle intervention versus face-to-face education and a paper brochure in patients with prostate cancer	Yes (improved aerobic exercise capacity in both groups, with higher increase in IG)	HRQoL	European Organization for Research and Treatment of Cancer Quality of Life Questionnaire-C30 (EORTC QLQ-C30)	No significant changes in global health status over time in either group. Significant improvements over time in all functional scales in both groups.	Significant group × time interaction in social functioning with higher increase in the IG.
				HRQoL	European Organization for Research and Treatment of Cancer Quality of Life Questionnaire-PR25 (EORTC-QLQ-PR25)	Significant improvement in the sexual functioning scale in CG only and in the urinary symptoms scale in both groups.	No SS difference
Radhakrishnan K et al., 2021 (29)	RCT	sensor-controlled digital game mobile app for self-management of medical condition versus sensors-only device in older patients with HF	Yes (improved self-care after 12 and 24 weeks in IG only; improved patient education on HF after 6, 12 and 24 weeks in both groups)	HRQoL	Kansas City Cardiomyopathy Questionnaire (KCCQ) – Items 13–15	Significant improvement in both groups from baseline to 12 and 24 weeks. Only in the CG, from baseline to 6 weeks.	*Between-group difference was not analyzed*
				Functional status	Kansas City Cardiomyopathy Questionnaire (KCCQ) – Items 1–12	Significant improvement from baseline to 24 weeks only in CG	*Between-group difference was not analyzed*
				Motivation for Self-management Behaviors	19-item Treatment Self-Regulation Questionnaire (TSRQ)	No SS changes	*Between-group difference was not analyzed*
				Self-efficacy	6-Item self-efficacy section of the Self-Care of Heart Failure Index	Significant improvement from baseline to 12 weeks only in the CG.	*Between-group difference was not analyzed*
Seok JW et al., 2022 (30)	Pre-post	AI-Driven Digital Health System for Promoting physical and psychological wellbeing in older adults	Yes (better nutritional status)	Depression	Geriatric Depression Scale Short Form (GDS), Korean version	No SS change	
				Fall fear	Tinetti Fall Efficacy Scale, Korean version	Significant improvement of anxiety related to fall^D^	
Wu R et al., 2024 (37)	Pre-post	mHealth app-based system for self-management of COPD	No SS change (severity of breathlessness)	HRQoL	Chronic Respiratory Disease Questionnaire (CRQ)	No SS change	
				HRQoL	Clinical COPD Questionnaire (CCQ)	No SS change	
				Self-Efficacy	COPD Self-Efficacy Scale	Significant decrease (worsening) of the Emotional Arousal Subscale score	
				Psychosocial impact of COPD	St George’s Respiratory Questionnaire	No SS change	
Yang WC et al., 2016 (31)	RCT	Non-Immersive VR Motion-Tracking system for balance training versus conventional home balance training in patients with PD	Yes (better balance and mobility in both groups at posttest and follow-up)	HRQoL	Parkinson’s Disease Questionnaire (PDQ-39)	Significantly improved HRQoL in both groups at posttest and follow-up	Not significant group and group × time effects.

Italics indicate data derived by the authors. ^A^For clarity, we grouped as “technology-related outcomes” those not classified as psychosocial. In most cases, these reflect the intended operational function of the technology. Main results are reported. ^B^Adopted by authors of the included papers or, when not specified, by relevant literature about the tool. ^C^Without the question “If you checked off any problems, how difficult have these problems made it for you to do your work, take care of things at home, or get along with other people?” ^D^The evidence should be treated with caution (see section 3.5.1 for details). CG, Control group; HRQoL, Health-related quality of life; IG, Intervention group; MCS, Mental Component Summary of the SF-36 (36-item Short Form Health Survey); mHealth, mobile health; PCS, Physical Component Summary of the SF-36 (36-item Short Form Health Survey); SS, Statistically significant.

All but 3 studies (26, 30, 39) considered Health-related quality of life (HRQoL). One study considered a broader health status, which includes aspects such as physiological, function, symptoms, functional impairment, HRQoL, QoL, satisfaction with relationships, and functional and mental status (36). The specific concepts of functional status (29) and psychosocial impact of the pathology (37) were also considered. Other concepts were related to perceived control, self-efficacy and motivation for self-management behavior (29, 37, 39).

The second most investigated outcome is depression (26–28, 30, 39), followed by fear of falling (27, 30, 40). Finally, QoL and loneliness were also considered (26, 28).

Regarding the measurements tools adopted, only one study (26) considered a general indicator of QoL, the short version of the quality of life assessment developed by the WHOQOL Group, the WHOQOL-BREF consisting of both physical, and psychological domains, but also the social and environment domains. As for HRQoL, two categories of indicators were used. The first category includes widely used generic questionnaires, such as the EuroQol-5D (27, 28, 36) which consists of a descriptive system (EQ-5D) and the EQ visual analogue scale, EQ-VAS, the Short Form Health Survey 36 (SF-36) (34, 40) and the WHO Disability Assessment Schedule (WHODAS) (27). On the other hand, disease-specific questionnaires were used, such as the European Organization for Research and Treatment of Cancer Quality of Life Questionnaire (33), the Kansas City Cardiomyopathy Questionnaire (29) or the Parkinson’s Disease Questionnaire (31), for instance.

In the case of depression, well-established questionnaires for clinical or geriatric populations were used, including the 9-item Patient Health Questionnaire (27) (PHQ-9), the Hospital Anxiety and Depression Scale (39), the Geriatric Depression Scale Short Form (28, 30), and also the proprietary tool PROMIS^®^ Item Bank v. 1.0 (26). Furthermore, in studies using HRQoL questionnaires, mental health dimensions were sometimes selected, such as the Mental Summary Component of the SF-36 (34, 40) and the anxiety/depression item of the EQ-5D (28).

Fear of falling was investigated using well-known tools assessing concerns about falling, such as the Falls Efficacy Scale-International (40), its variant the Iconographical Falls Efficacy Scale (27), and the Tinetti Falls Efficacy Scale (30).

Despite the fact that numerous studies have investigated the same outcome—such as depression and HRQoL—, the heterogeneity in definitions, methodologies, and measurement approaches prevents a reliable quantitative synthesis of the impact of technologies on this outcome. In the case of depression, for example, these measurement tools range from single-item measures (e.g., EQ-5D), to symptom-specific scales (e.g., PHQ-9), to composite indices (e.g., Mental Component Summary of the SF-36). Moreover, the intended use of these instruments differs significantly: some are designed for screening (PHQ-9), others for monitoring changes over time (Mental Component Summary of the SF-36), and others for evaluating health utility (EQ-5D). For this reason, in the following sub-sections, we provide a reasoned synthesis of the evidence gathered.

### 3.5 Reported evidence on the impact of using technologies on psychosocial outcomes

The analysis of the evidence is organized according to a 2 × 2 framework, which integrates findings related to both technology performance and psychosocial outcomes (see Table 4). This framework functions as a formal interpretative tool designed to assess the reliability of the emerging evidence relevant for this review, while also identifying potential methodological limitations that may have influenced certain results or undermined others. It is important to emphasize that no causal relationship between the goodness or failure of technological performance and psychosocial impact is implied. Arguments of this nature lie beyond the scope of this review. The specific findings concerning technology performance (e.g., gait speed, exacerbation-free weeks, individual fall risk, etc.) are presented as reported by the original authors, without any additional evaluation or critical appraisal.

Only one (35) of the studies does not fit this scheme because the only part of interest for this review is limited to the impact on psychosocial outcomes in a subgroup for which technological performance is not detailed? (Table 4).

#### 3.5.1 When changes in both technology-related and psychosocial outcomes are both significant

A subset of studies reported significant changes in technology-related outcomes and in psychosocial outcomes (28–31, 33, 34, 40). HRQoL outcomes were considered in all of these studies except Seok et al. (30). Improvements over time in HRQoL were generally not limited to the intervention groups (29, 31, 33, 34). When comparing changes between different groups, the results are very heterogeneous: greater improvements in the intervention groups (28, 34), no difference between groups (31), and significant time-group interactions (33, 40). The evidence reported in Lim et al. (28) regarding the EQ-5D items, however, should be considered with great caution as the statistical approach used does not align with the categorical nature of the measure. The Stuart-Maxwell test for marginal homogeneity would have been more appropriate, for example.

Note that the study by Ginis et al. (40) is the only one to also considering maintenance after post-test. In this study, the significance of the interaction between group and time comes from a worsening of the physical health score of the SF-36 in the control group at follow-up after an improvement at post-test rather than from an improvement in the intervention group, where there are no changes over time. Even in the studies by Yang et al. (31) and Radhakrishnan et al. (29) a follow-up measure is formally considered, but this measure is compared only with the baseline data, and therefore it is not possible to evaluate maintenance.

Radhakrishnan et al. (29) also considered functional status and self-care/management concepts in their study. Significant results were mainly obtained in the control group. Compared to the baseline, heart failure self-management improved at the clinical level in both groups at 6, 12 and 24 weeks. However, motivation for self-management behaviors and self-efficacy did not substantially change, maybe due to high knowledge of HF self-management at baseline in both groups.

Two studies (28, 30) also considered depression in older adults living alone. No pre-post changes were found in Seok et al. (30). In the study by Lim et al. (28), only between group difference was statistically analyzed and was found significant (indicating a significantly greater reduction in depression in the intervention group vs. control group), while the within-group change over time was not evaluated for statistical significance and was only reported at a descriptive level. However, the summary data on Depression reported in their Table 4 appear to be inconsistent with the description provided in the main text of the adopted scale, the short form of the Korean version of the GDS. In the group that followed the intervention program with the social robot PIO (28), loneliness was reduced to a greater and statistically significant extent than in the control group and we could verify by ourselves that the within-group variation is significant in the intervention group and not in the control group.

Fear of falling was considered in two studies (30, 40). Having used or not an AAL system in subjects with Parkinson’s Disease did not have a statistically significant impact on the fear of falling, which did not change over time in any group (40). Seok et al. (30) in their pre-post study of older adults living alone reported in the text that *use of the system significantly improved participants’ fall-related anxiety*, although they reported an opposite result in their Table 3.

#### 3.5.2 When changes in technology-related outcomes are not significant while changes in psychosocial outcomes are

The case in question occurs only in the study by Wu et al. (37) and only because of one statistically significant result among the many that were not. In this pre-post study on subjects with chronic obstructive pulmonary disease, a self-management application for smartphones and smartwatches did not reduce the severity of breathlessness and its impact on daily activities. Therefore, the lack of change in all but one of the psychosocial outcomes also appears to be quite consistent. In relation to the worsening in the emotional arousal subscale of the COPD Self Efficacy Scale following the use of the app, the authors suggest as possible reasons a statistical artifact, a declining trajectory of self-efficacy in patients with COPD over time as reported in literature, and also the dissatisfaction of a high expectation on the use of the technology.

#### 3.5.3 When changes in technology-related outcomes are significant while changes in psychosocial outcomes are not

In some of the studies (26, 27, 32, 39), significant changes in certain technology-related outcomes were reported, but no significant changes were observed on psychosocial outcomes. The psychosocial outcome measurement tools such as the CAS, EQ-5D, HADS, OHIP-14, PHQ-9, PROMIS®, WHODAS, and WHOQOL-BREF were originally designed and validated for a wide range of purposes that often differ from the aims for which they were applied in the studies. For instance, the EQ-5D was developed primarily for cost-utility analyses (41), while the PHQ-9 and HADS are commonly used as diagnostic screening tools. The remaining tools were designed for longitudinal monitoring within specific populations. As a result, these tools may fail to detect changes after the intervention, as they are generally designed to capture broader shifts in health status, rather than changes directly linked to specific and limited interventions.

#### 3.5.4 When changes in technology-related and psychosocial outcomes are both non-significant

Keogh et al. (38) and Boer et al. (36) evaluated the use of technologies to support self-management of a disease (COPD and HF, respectively). In both studies, the technology did not lead to any statistically significant changes in any of the outcomes evaluated. In the pre-post study of Keogh et al. (38) the authors highlight that moderate levels of both self-care ability and quality of life at the baseline may be the reason for the lack of significant improvement. Something similar seems to explain the lack of differences between the intervention and control groups in Boer et al. (36). As the authors themselves note, it was the experiment itself that leveled the participants’ ability to self-manage their disease, by offering both the intervention and control groups a short educational session on recognition and treatment of exacerbations before the start of the study.

No condition-specific (e.g., chronic obstructive pulmonary disease, Parkinson’s disease, and heart failure) trend toward enhanced psychosocial benefit emerged from the included studies.

## 4 Discussion

This review contributes to a growing understanding of whether using AAL technologies is associated with positive changes in psychosocial outcomes in older adults and offers an opportunity to reflect on methodological approaches.

### 4.1 Population and technology

Some of the studies are focused on specific health conditions while others did not. Studies addressing chronic conditions tend to prioritize technological solutions aimed at clinical monitoring, self-management, remote consultation, or symptom alleviation. By contrast, studies involving otherwise healthy older adults focus more on lifestyle promotion, emotional support, and cognitive stimulation—objectives that resonate with a broader vision of longevity and independent living. Notably, technologies designed to guide and monitor physical exercise programs are found across both typologies of studies, confirming the centrality of physical activity in healthy aging (42).

### 4.2 Psychosocial outcomes and older adults

A central insight from this review is that a conscious reference to the dimensions of psychosocial well-being relevant to the impacts of the proposed technology is not evident. The choice of the targeted psychosocial outcome is implicitly entrusted to the instrument used to measure it but, apparently, without awareness, and what to measure often appears guided by the popularity of certain instruments rather than by a thoughtful evaluation. This has repercussions on the discussion of the findings. Seok et al. (30), for instance, conclude about reduced depressive feelings in association with the ability of the system to motivate older adults to spend time outdoors and increase physical activity. However, this conclusion is not substantiated by the results obtained from the Korean version of the short form GDS (GDS-SF). This screening tool is not designed to detect subtle mood fluctuations or “depressive feelings,” only marginally addresses behaviors related to going outdoors, and does not indicate whether individuals actually engage in such behaviors. Moreover, it fails to capture any mood improvements that may result from these activities. Accurate interpretation of findings depends on using appropriate instruments that are sensitive to the specific psychosocial outcomes being evaluated in the targeted populations. In this case, a more targeted measure of positive and negative mood [e.g., positive and negative affect scale (43)] would have been more appropriate than a general depression screener.

Because technologies aimed at supporting adults with certain diseases are prevalent, we found widespread use of HRQoL measures. However, the impact of the use of technologies like those considered here should be better considered from a more comprehensive view, including other dimensions of life, such as social relationships, remaining mentally active, and having meaningful goals (44). This broader perspective is reflected in the WHO (World health organization) definition of *Healthy Ageing*, which, consistent with the conceptual transition underpinning the longevity culture (5), focuses not on the absence of diseases but on maintaining *functional ability* to meet basic needs, remain mobile, sustain relationships, and contribute to society (45).

### 4.3 Toward more interpretable and actionable evidence

A critical issue concerns the statistical treatment of psychosocial data. In several studies, the statistical tests applied did not appear well suited to the nature or distribution of the data. This included, for instance, the use of parametric methods with ordinal or non-normally distributed data, or the treatment of categorical outcomes as continuous variables. This may compromise the validity of reported findings and limit the interpretability of psychosocial effects.

In some of the included works, results synthesis was hampered by shortcomings in the statistical analyses or even by the improper use of psychosocial indicators. For example, some works (36, 39, 40) reported total scores not foreseen by the instrument’s original validation, while others (26, 34) analyzed sub-dimensions of scales individually, even against recommendations of the user manuals. Using validated tools, as in the selected studies, is certainly important but not enough: if they are applied in contexts other than those for which they were designed and if the data are analyzed improperly, the results obtained remain questionable. Psychosocial indicators, such as questionnaires and self-reports, typically rely on assumptions about latent factors or traits that are tested during the validation. Using an instrument outside its validation scope, such as analyzing a total score when only specific sub scales have been validated, undermines both the validity and statistical reliability of the results (46). For example, using a screening tool to monitor the effects of a technology is like using a smoke detector to assess air quality. Likewise, aggregating total scores from a multidimensional questionnaire is like averaging the side lengths of a polygon to estimate its area.

In some cases, issues of unclear reporting further compromised interpretability with the emerging of discrepancies between the declared instrument and the reported scores (28), ambiguity in the scoring method (26), or missing information on data transformation (27). Clear documentation of the version used and adherence to validated scoring methods are essential to ensure the reliability and comparability of findings.

As regards the statistical methods adopted, the major criticality concerns the assumption of Gaussianity. Statistical analysis of SF-36 data, for instance, requires nonparametric methods (47). In fact, the empirical distributions of this type of indicators are often asymmetric, suggesting that in small sample sizes the Gaussian-based inference (*t*-test, ANOVA) is not appropriate. Additionally, inferential methods for continuous variables cannot be applied to categorical data, such as the single Likert-scale item subdimension of some indicators (28).

Finally, the presentation of the collected data often follows the scheme of comparison between two independent groups, preventing the replicability of the tests conducted and the “eyeball” evaluation of the true variable of interest, the within-person differences.

In almost all studies, findings were discussed in terms of statistical significance (*p* values), with limited consideration of the relevance of observed changes.

Another issue is when indicators show a fairly good psychosocial condition even before treatment, it can also be expected that this condition may not show significant changes due to the treatment (ceiling effect). For instance, Boer et al. (36) found no statistically significant difference in CCQ levels between intervention and control groups. Both groups showed high baseline values on this HRQoL indicator, suggesting a good health status at baseline. The situation of participants with critical health conditions is the opposite. Critical levels of psychosocial indicators at baseline may prevent the detection of beneficial aspects of the technology, as could have been occurred in the study of Liljeroos (39) on patients with moderate to severe HF and reporting more than mild symptoms of anxiety at baseline.

Furthermore, if a good familiarity with the technology contributed to the decision to participate in the trial, the subjects may also have a lower perception of the effect of the technology because of the diminishing returns effect.

None of the included studies explicitly addressed participant predispositions that may affect both engagement and sensitivity to psychosocial outcomes. These factors should warrant consideration in both study design and interpretation.

Several studies included features in their design or implementation that may have introduced unintended influences on outcomes or participation. These contextual effects arise from control conditions, researcher involvement, or informal caregiving dynamics. In Boer et al. (36), participants in the control group were asked to follow their usual care but were also given a paper exacerbation plan, as recommended by current Dutch COPD guidelines. The authors of the study noted that this turned into a kind of intervention since many of the subjects in the control group had never received a care plan before. This increased attention to the behaviors to be adopted for one’s own care may have led to the absence of differences in effects between treatment and control. This suggests that if you plan an RCT and expect the control group to follow the usual care/ or usual lifestyle, you need to understand what this means for the study subjects. In addition, any other initiatives not strictly related to testing the technology need to be carefully evaluated for their possible effects on the measure of effectiveness.

Although many interventions were implemented in settings intended to reflect real-life conditions, none were conducted in the complete absence of interpersonal interaction. In trials relying on self-reported psychosocial outcomes, even limited contact with study personnel may unintentionally influence participant perceptions or behaviors [Hawthorne effect (48)]. Yet no studies reported strategies to isolate such contextual effects—such as, for example, control groups matched for level of contact but without active intervention, or *post hoc* assessments of perceived attention received. The absence of such measures limits confidence in attributing observed improvements to the intervention itself, rather than to participants’ responsiveness to the study context.

Trials involving participants with complex health conditions or requiring informal care support may inadvertently impose burdens that affect adherence or retention. In particular, regular in-person or remote interactions with study staff—such as nurses, therapists, or researchers—may create logistical challenges or disrupt daily routines. While such presence is often necessary for intervention delivery or monitoring, it also constitutes an additional layer of engagement that is not always acceptable or sustainable. Yang et al. (31), for example, in their study on individuals affected by PD reported that some patients declined to enroll because of the perceived inconvenience of regular home visits.

All these methodological and contextual weakness may lead to what Sen et al. (49) brilliantly highlighted: *ambiguity remains as to whether technology use predicted social connectedness and better health outcomes, or whether already existing social connections and better health in the elderly predicted more technology use*. Technology for older adults depends not only on innovation, but on the production of reliable, interpretable, and actionable evidence that can inform both practice and policy.

### 4.4 Older adults as agents of technological innovation

Despite growing attention to user perspectives, evident in the eight studies that investigated experience, acceptability and usability (26, 27, 29, 30, 36, 38–40), only three studies (36, 37, 40) proposed technologies developed in collaboration with representatives of future users. This shortfall is not merely procedural; it reflects a broader, often implicit, framing of aging as a static condition—a target for interventions or a set of problems to be solved (50). Recent literature challenges this view, describing the relationship between aging and technology as dynamic and bidirectional (51). This became particularly evident during the COVID-19 pandemic, which accelerated the uptake of digital platforms among older adults, not only for communication but also for sustaining meaningful social, cultural and civic engagement, with positive psychosocial consequences (28, 52). Because aging is a complex and ever-evolving phenomenon, engaging older adults in the design and not just testing of technology products and services through a co-design approach is a challenging but valuable effort. A person-centered, co-design approach should take into account that older people’s use of technology can change their perception of their role. Recognizing this dynamic can bring the co-design approach to its best expression and trigger further innovation (50, 53). A useful way to conceptualize the role of older adults in technological innovation is through a graduated typology of engagement. For example, at the consultative level, older adults provide feedback on prototypes, ideas, or usability issues, typically through interviews, surveys, or focus groups (54). This stage ensures that design decisions are informed by lived experience, although their influence remains partially external to the development process. At the participatory level, older adults are involved more substantially in shaping design priorities, participating in iterative testing cycles, and advising on features that align with daily routines, preferences, and constraints (55, 56). Embedding AAL research within such a structured engagement framework can support the development of technologies that are more acceptable, meaningful, and responsive to the evolving realities of aging.

### 4.5 Strengths and limitations

The heterogeneity of the included studies, particularly in assessment methods, rendered a meta-analysis inappropriate. In addition, as discussed earlier, the overall quality of the evidence was moderate. Another limitation is that the review protocol was not prospectively registered and this can raise concerns about selective decisions. However, an internally defined protocol guided the entire review process; moreover, the search strategy and the list of studies that might appear to meet the inclusion criteria but which were excluded are provided as Supplementary material to enhance transparency and reproducibility.

A key strength of this review is its systematic synthesis of evidence on the impact of AAL technologies on psychological well-being. Our findings also highlight the need for a standardized framework of psychosocial well-being dimensions and corresponding measures capable of capturing how AAL technologies support older adults in meeting basic needs, maintaining mobility, sustaining relationships, and contributing to society. These insights can inform methodological improvements and guide the generation of more interpretable and actionable evidence.

### 4.6 Conclusion

This review highlights the positive impact of Active and Assisted Living technologies on psychosocial well-being in older adults and the need for refined methodological approaches. While technologies developers have started to consider the psychosocial impact in addition to technology-related outcomes, this study confirms that a conceptual framework within which to place the development, testing and validation of technologies for daily living is still in progress. An overall agreed reference to the dimensions of psychosocial well-being relevant to older adults should be found together with measures that collects the capacity of maintaining functional ability to meet basic needs, remaining mobile, sustaining relationships, and contributing to society.

Finally, our review represents an opportunity to highlight methodological approaches and provide suggestions for a more interpretable and actionable evidence.

## Data Availability

The original contributions presented in the study are included in the article/Supplementary material, further inquiries can be directed to the corresponding author.
