# Supplementary material for: Effect of Active and Assisted Living technologies on psychosocial well-being in older adults: systematic review
Source: Front Public Health. 2026 Jan 8;13:1717154. doi: 10.3389/fpubh.2025.1717154 (PMC12823859; doi:10.3389/fpubh.2025.1717154)
Supplement: Supplementary file 1 [file Supplementary_file_1.docx]

**SEARCH STRATEGY**

**SOURCES:**

- **EBM Reviews - Cochrane Central Register of Controlled Trials**
- **Ovid Healthstar**
- **Ovid MEDLINE(R) and Epub Ahead of Print, In-Process, In-Data-Review & Other Non-Indexed Citations, Daily and Versions**

|  | String |
| --- | --- |
| P | A: exp Home Care Services/ or Home Care Service*.mp. or Domiciliary Care.mp. or Ambient Intelligence.mp. or exp Monitoring, Ambulatory/ or Monitoring, Ambulatory.mp. or Outpatient Monitor*.mp. or Ambient Intelligence/ or Assisted Living.mp. or Health Services for the Aged/ or Health Services.mp. or Assisted Living Facilities/ or smart.mp. or Homes for the Aged/ or (Residential adj Facilit*).mp. or Home*.mp. or House*.mp. or environment*.mp. or Health Services for the Aged/ or Independent Living/ or Independent Living.mp or aging in place.mp or ageing in place.mp |
|  | B: exp Aged/ or elder*.mp. or (old* adj (person* or adult* or people)).mp. or Above 65 years.mp. or senior*.mp. or aging.mp. or ageing.mp. |
| I | Remote Sensing Technology/ or exp Information Systems/ or Mobile Applications/ or exp Wearable Electronic Devices/ or ((remote or wearable or sensing or mobile or information or conversational or gaming) adj (technolog* or system* or device* or tool* or application*)).mp. or ((companion or assistive or social) adj Robot*).mp. or ((Fitness or Activity) adj Tracker*).mp. or exp Virtual Reality/ OR Virtual Reality.mp or Internet of Things.mp. or IoT.mp or exp telemedicine/ or telemedicine.mp OR telemonitor*.mp |
| C | NA |
| O | exp "Quality of Life"/ or (well-being or wellbeing).mp. or emotional health.mp. or mental health/ or psychosocial.mp. or (Limitation adj3 Activit*).mp. or Self efficacy/ or self-efficacy.mp. or exp self-management/ or self-management.mp. or Empowerment/ or empowerment.mp. or exp activities of daily living/ or (activit* adj3 (daily living)).mp. or ADL.mp. or Community Integration/ or (communit* adj3 integration).mp. or social participation.mp. or exp self concept/ or self-esteem.mp. or self-perception.mp. or self confidence.mp. or psychological well-being/ or Psychosocial Functioning/ or Psychosocial.mp |
| S | Exp clinical trial/ or exp clinical trial as topic/ or multicenter study/ or Multicenter Studies as Topic/ or Controlled Before-After Studies/ or clinical trials as topic.sh. or randomized controlled trial.pt. or controlled clinical trial.pt. or clinical trial.pt or evaluation study.pt or multicenter study.pt or pragmatic clinical trial.pt. or comparative study.pt. or case reports.pt or clinical study or random*.ti,ab,kw or sham.ti,ab,kw or trial.ti,ab,kw. Or control*.ti,ab,kw. Or compar*.ti,ab,kw. |
|  | P_A_ AND P_B_ AND I AND O AND S |

**SOURCE: IEEE**

[**https://ieeexplore.ieee.org/search/**](https://ieeexplore.ieee.org/search/)

(

("Mesh_Terms":Community Health Services) OR ("Mesh_Terms": Home Care Services, Hospital-Based) OR ("Mesh_Terms": Home Health Nursing) OR ("Mesh_Terms": Home Nursing) OR ("All Metadata":“Home care”) OR ("All Metadata":“Domiciliary care”) OR ("All Metadata":“Ambient intelligence”) OR ("Mesh_Terms": Monitoring, Ambulatory) OR ("Mesh_Terms":Blood Pressure Monitoring, Ambulatory) OR ("Mesh_Terms": Electrocardiography, Ambulatory) OR ("All Metadata":“Outpatient monitor*”) OR ("All Metadata":“Assisted living”) OR ("Mesh_Terms": Health Services for the Aged) OR ("Mesh_Terms": Assisted Living Facilities) OR ("All Metadata":“Living facilit*”) OR ("All Metadata":“Assisted living*”) OR ("All Metadata":“Smart”) OR ("All Metadata":“hous*”) OR ("All Metadata":“environment*”) OR ("Mesh_Terms": Homes for the Aged) OR ("All Metadata":“Residential Care facilit*”) OR ("Mesh_Terms": Health Services for the Aged) OR ("Mesh_Terms": Independent Living)

OR ("All Metadata":“Independent living”) OR ("All Metadata":“Aging in place”) OR ("All Metadata":“Ageing in place”)

)

AND

(

("Mesh_Terms":Aged) OR ("Mesh_Terms":Aged, 80 and over) OR ("Mesh_Terms":Centenarians) OR ("Mesh_Terms":Nonagenarians) OR

("Mesh_Terms":“Octogenarians”) OR ("All Metadata":“Elder”) OR ("All Metadata":“Elders”) OR ("All Metadata":“Elderly”) OR ("All Metadata":“Old persons”) OR ("All Metadata":“Old person”) OR ("All Metadata":“Old people”) OR ("All Metadata":“Old adult”) OR ("All Metadata":“senior” )  OR ("All Metadata":“Ageing”) OR ("All Metadata":“Aging”)

)

AND

(

("All Metadata":“Sensing Technology”) OR ("Mesh_Terms": Remote Sensing Technology) OR ("Mesh_Terms": Information Systems) OR ("Mesh_Terms":Big data) OR ("Mesh_Terms": Community Networks) OR ("Mesh_Terms": Decision Support Systems, Clinical) OR ("Mesh_Terms": Health Information Systems) OR ("Mesh_Terms": Medical Records Systems, Computerized) OR ("Mesh_Terms": Mobile Applications) OR ("All Metadata":“Companion robot”) OR ("All Metadata":“Assistive robot”) OR ("All Metadata":“Social robot”) OR ("Mesh_Terms": Wearable Electronic Devices) OR ("Mesh_Terms": Fitness Trackers) OR ("All Metadata":“Fitness tracker”) OR ("All Metadata":“Activity tracker”) OR ("Mesh_Terms": Hearing Aids) OR ("Mesh_Terms": Smart Glasses) OR ("All Metadata":“Wearable Technology”) OR ("All Metadata":“Wearable Device”) OR ("All Metadata":“Conversational technology”) OR ("All Metadata":“Gaming technology”) OR ("Mesh_Terms": Virtual Reality) OR ("Mesh_Terms": Haptic Technology) OR ("All Metadata":“Virtual Reality”) OR ("All Metadata":“Internet of Things”) OR ("All Metadata":“IoT”) OR ("Mesh_Terms":Telemedicine) OR ("Mesh_Terms": Telerehabilitation) OR ("Mesh_Terms": Remote Consultation) OR ("Mesh_Terms": Telenursing) OR ("All Metadata":“Telemedicine”)   OR ("All Metadata":“Telemonitor”)  OR ("All Metadata":“Telemonitoring”)

)

AND

(

("Mesh_Terms": Quality of Life) OR ("Mesh_Terms": Psychological Well-Being) OR ("All Metadata":“Psychological well-being”) OR ("All Metadata":“Well-being”) OR ("All Metadata":“Wellbeing”) OR ("All Metadata":“Emotional health”) OR ("Mesh_Terms": mental health) OR ("All Metadata":“Mental health”) OR ("All Metadata":“Psychosocial”) OR ("All Metadata":“Psycho-social”)  OR ("All Metadata":“Limitation of Activity”) OR ("All Metadata":“Limitation of Activities”) OR ("Mesh_Terms": Self efficacy) OR ("All Metadata":“Self-efficacy”) OR ("Mesh_Terms": self-management) OR ("All Metadata":“Self-management”) OR ("Mesh_Terms": Empowerment) OR ("All Metadata":“Empowerment”) OR ("Mesh_Terms": Activities of Daily Living) OR ("Mesh_Terms": Independent Living) OR ("Mesh_Terms": Self Care) OR ("Mesh_Terms": Self-Neglect) OR ("Mesh_Terms": Social Participation) OR ("All Metadata":“Social participation”) OR ("Mesh_Terms": Functional Status) OR ("All Metadata":“Daily living”) OR ("All Metadata":“ADL”) OR ("Mesh_Terms": Community Integration) OR ("All Metadata":“Community integration” )   OR ("All Metadata":“Social integration”) OR ("Mesh_Terms": self concept) OR ("Mesh_Terms": Self-Assessment) OR ("Mesh_Terms": Self Efficacy) OR ("Mesh_Terms": Sense of Coherence) OR ("Mesh_Terms": Social Comparison) OR ("All Metadata":“Self-concept” )   OR ("All Metadata":“Self-esteem”) OR ("All Metadata":“Self-perception”) OR ("All Metadata":“Self-confidence”) OR ("Mesh_Terms": Psychosocial Functioning) OR ("All Metadata":“Psychosocial Function”) OR ("All Metadata":“Psychosocial Function”) OR ("All Metadata":“Psychosocial Functioning”) OR ("All Metadata":“Psychosocial” )

)

**SOURCE: APA PsychArticles**

[**https://psycnet.apa.org/search/**](https://psycnet.apa.org/search/)

(

**MeSH:** Home Care Services OR **Any Field:** “Home Care Service*” OR **Any Field:** “Domiciliary Care” OR **Any Field:** “Ambient” OR **MeSH:** Monitoring, Ambulatory OR **Any Field:** “Ambulatory Monitor*” OR **Any Field:** “Outpatient Monitoring” OR **MeSH:** “Ambient Intelligence” OR **Any Field:** “Assisted Living” OR **MeSH:** Health Services for the Aged OR **MeSH:** Assisted Living Facilities OR **Any Field:** “Living Facilit*” OR **Any Field:** “Assisted Facilit*” OR **Any Field:** “smart” OR **Any Field:** “house*” OR **Any Field:** “environment*” OR **MeSH:** Homes for the Aged OR **Any Field:** “Residential Facilit*” OR **Any Field:** “Residential Care Facilit*” OR **Any Field:** “Home*” OR **MeSH:** Health Services for the Aged OR **MeSH:** Independent Living OR **Any Field:** “Independent Living” OR **Any Field:** “aging in place” OR **Any Field:** “ageing in place”

)

AND

(

**MeSH:** Aged OR **Any Field:** elder* OR **Any Field:** “old person*” OR **Any Field:** “old people” OR **Any Field:** “old adult*” OR **Any Field:** “senior*” OR **Any Field:** “ageing” OR **Any Field:** “aging”

)

AND

(

**MeSH:** Remote Sensing Technology OR **Mesh:** Information Systems OR **MeSH:** Mobile Applications OR **Any Field:** “companion robot*” OR **Any Field:** “assistive robot*” OR **Any Field:** “social robot*” OR **MeSH:** Wearable Electronic Devices OR **MeSh:** Virtual Reality OR **Any Field:** “Virtual Reality” OR **Any Field:** “Internet of Things” OR **Any Field:** “IoT” OR **MeSh:** Telemedicine OR **Any Field:** Telemedicine OR **Any Field:** Telemonitor* OR **Any Field:** “Remote Technolog*” OR **Any Field:** “Wearable Technolog*” OR **Any Field:** “Sensing Technolog*” OR **Any Field:** “Mobile Technolog*” OR **Any Field:** “Information Technolog*” OR **Any Field:** “Conversational Technolog*” OR **Any Field:** “Gaming Technolog*” OR **Any Field:** “Remote System*” OR **Any Field:** “Wearable System*” OR **Any Field:** “Sensing System*” OR **Any Field:** “Mobile System*” OR **Any Field:** “Information System*” OR **Any Field:** “Conversational System*” OR **Any Field:** “Gaming System*” OR **Any Field:** “Remote Device*” OR **Any Field:** “Wearable Device*” OR **Any Field:** “Sensing Device*” OR **Any Field:** “Mobile Device*” OR **Any Field:** “Information Device*” OR **Any Field:** “Conversational Device*” OR **Any Field:** “Gaming Device*” OR **Any Field:** “Remote Tool*” OR **Any Field:** “Wearable Tool*” OR **Any Field:** “Sensing Tool*” OR **Any Field:** “Mobile Tool*” OR **Any Field:** “Information Tool*” OR **Any Field:** “Conversational Tool*” OR **Any Field:** “Gaming Tool*” OR **Any Field:** “Remote Application*” OR **Any Field:** “Wearable Application*” OR **Any Field:** “Sensing Application*” OR **Any Field:** “Mobile Application*” OR **Any Field:** “Information Application*” OR **Any Field:** “Conversational Application*” OR **Any Field:** “Gaming Application*” OR **Any Field:** “Fitness Tracker*”

)

**SOURCE: SCOPUS**

(

TITLE-ABS-KEY ( “Home care service*” )  OR  TITLE-ABS-KEY ( “Domiciliary care” )  OR  TITLE-ABS-KEY ( “Ambient intelligence” )  OR  TITLE-ABS-KEY ( “Ambulatory monitor*” )  OR  TITLE-ABS-KEY ( “Outpatient monitor*” )  OR  TITLE-ABS-KEY ( “Assisted living” )  OR  TITLE-ABS-KEY ( “Living facilit*” )  OR  TITLE-ABS-KEY ( “Assisted facilit*” )  OR  TITLE-ABS-KEY ( “Smart” )  OR  TITLE-ABS-KEY ( “house*” )  OR  TITLE-ABS-KEY ( “environment*” )  OR  TITLE-ABS-KEY ( “Residential Care facilit*” )   OR  TITLE-ABS-KEY ( “home*” )   OR  TITLE-ABS-KEY ( “Independent living” )  OR  TITLE-ABS-KEY ( “Aging in place” )  OR  TITLE-ABS-KEY ( “Ageing in place”)

)

AND

(

TITLE-ABS-KEY ( Elder* )  OR  TITLE-ABS-KEY ( “Old person*” )  OR  TITLE-ABS-KEY ( “Old people” )  OR  TITLE-ABS-KEY ( “Old adult*” )  OR  TITLE-ABS-KEY ( ageing ) OR  TITLE-ABS-KEY ( aging ) OR  TITLE-ABS-KEY ( senior* )

)

AND

(

TITLE-ABS-KEY ( “Companion robot*” )  OR  TITLE-ABS-KEY ( “Assistive robot*” )  OR  TITLE-ABS-KEY ( “Social robot*” )   OR  TITLE-ABS-KEY ( “Virtual Reality” )  OR  TITLE-ABS-KEY ( “Internet of Things” )  OR  TITLE-ABS-KEY ( IoT )  OR  TITLE-ABS-KEY ( Telemedicine )   OR  TITLE-ABS-KEY ( Telemonitor* )  OR TITLE-ABS-KEY ( “Remote Technolog*”)  OR  TITLE-ABS-KEY ( “Wearable Technolog*”)  OR   TITLE-ABS-KEY ( “Sensing Technolog*”)  OR   TITLE-ABS-KEY ( “Mobile Technolog*”)  OR  TITLE-ABS-KEY ( “Information Technolog*”)  OR   TITLE-ABS-KEY ( “Conversational Technolog*”)  OR   TITLE-ABS-KEY ( “Gaming Technolog*”)  OR   TITLE-ABS-KEY ( “Remote System*”)  OR   TITLE-ABS-KEY ( “Wearable System*”)  OR   TITLE-ABS-KEY ( “Sensing System*”)  OR   TITLE-ABS-KEY ( “Mobile System*”)  OR   TITLE-ABS-KEY ( “Information System*”)  OR   TITLE-ABS-KEY ( “Conversational System*”)  OR   TITLE-ABS-KEY ( “Gaming System*”)  OR   TITLE-ABS-KEY ( “Remote Device*”)  OR   TITLE-ABS-KEY ( “Wearable Device*”)  OR   TITLE-ABS-KEY ( “Sensing Device*”)  OR   TITLE-ABS-KEY ( “Mobile Device*”)  OR   TITLE-ABS-KEY ( “Information Device*”)  OR   TITLE-ABS-KEY ( “Conversational Device*”)  OR   TITLE-ABS-KEY ( “Gaming Device*”)  OR   TITLE-ABS-KEY ( “Remote Tool*”)  OR   TITLE-ABS-KEY ( “Wearable Tool*”)  OR   TITLE-ABS-KEY ( “Sensing Tool*”)  OR   TITLE-ABS-KEY ( “Mobile Tool*”)  OR   TITLE-ABS-KEY ( “Information Tool*”)  OR   TITLE-ABS-KEY ( “Conversational Tool*”)  OR   TITLE-ABS-KEY ( “Gaming Tool*”)  OR   TITLE-ABS-KEY ( “Remote Application*”)  OR   TITLE-ABS-KEY ( “Wearable Application*”)  OR   TITLE-ABS-KEY ( “Sensing Application*”)  OR   TITLE-ABS-KEY ( “Mobile Application*”)  OR   TITLE-ABS-KEY ( “Information Application*”)  OR   TITLE-ABS-KEY ( “Conversational Application*”)  OR   TITLE-ABS-KEY ( “Gaming Application*”)  OR   TITLE-ABS-KEY  ( “Fitness Tracker*”)

)

AND

(

TITLE-ABS-KEY ( Well-being )  OR  TITLE-ABS-KEY ( Wellbeing )  OR  TITLE-ABS-KEY ( “Emotional health” )  OR  TITLE-ABS-KEY ( “Mental health” )  OR  TITLE-ABS-KEY ( Psychosocial )  OR  TITLE-ABS-KEY ( Psycho-social )  OR  TITLE-ABS-KEY ( “Limitation of Activit*” )  OR  TITLE-ABS-KEY ( Self-efficacy )  OR  TITLE-ABS-KEY ( Self-management )  OR  TITLE-ABS-KEY ( Empowerment )  OR  TITLE-ABS-KEY ( “Daily living” )  OR  TITLE-ABS-KEY ( ADL )  OR  TITLE-ABS-KEY ( “Community integration” )   OR  TITLE-ABS-KEY ( “Social integration” )  OR  TITLE-ABS-KEY ( “Social participation” )  OR  TITLE-ABS-KEY ( Self-concept )   OR  TITLE-ABS-KEY ( Self-esteem )  OR  TITLE-ABS-KEY ( Self-perception)  OR  TITLE-ABS-KEY ( Self-confidence )  OR  TITLE-ABS-KEY ( “Psychological well-being” )  OR  TITLE-ABS-KEY ( “Psychosocial Function*” )  OR  TITLE-ABS-KEY ( Psychosocial )

)

AND

(

ALL ( “Clinical trial” )  OR  ALL  ( “Multicenter study” )  OR  ALL  ( “Controlled trial” )  OR  ALL  ( “Randomized trial” )  OR  ALL  ( “Clinical trial” )  OR  ALL  ( “Comparative study” )  OR  ALL  ( Random* )  OR  ALL  ( Sham )  OR  ALL  ( Controlled Before-After Studies)       OR  ALL  ( evaluation study)       OR  ALL  ( multicenter study)       OR  ALL  ( case reports)       OR  ALL  ( clinical study )

)

AND

(

LIMIT-TO ( DOCTYPE ,  ”ar” )

)

AND

(

EXCLUDE ( SUBJAREA, “BIOC” ) OR EXCLUDE ( SUBJAREA, “ARTS” ) OR EXCLUDE ( SUBJAREA, “AGRI” ) OR EXCLUDE ( SUBJAREA, “PHYS” ) OR EXCLUDE ( SUBJAREA, “CHEM” ) OR EXCLUDE ( SUBJAREA, “CENG” ) OR EXCLUDE ( SUBJAREA, “MATE” ) OR EXCLUDE ( SUBJAREA, “MATH” )

)

**SOURCE: WoS**

(

ALL=( “Home care service*” )  OR  ALL=( “Domiciliary care” )  OR  ALL=( “Ambient intelligence” )  OR  ALL=( “Ambulatory monitor*” )  OR  ALL=( “Outpatient monitor*” )  OR  ALL=( “Assisted living” )   OR  ALL=( “Living facilit*” )  OR  ALL=( “Assisted facilit*” )  OR  ALL=( “Smart” )  OR  ALL=( “house*” )  OR  ALL=( “environment*” )  OR  ALL=( “Residential Care facilit*” )   OR  ALL=( “home*” )   OR  ALL=( “Independent living” )  OR  ALL=( “Aging in place” )  OR  ALL=( “Ageing in place”)

)

AND

(

ALL=( Elder* )  OR  ALL=( “Old person*” )  OR  ALL=( “Old people” )  OR  ALL=( “Old adult*” )  OR  ALL=( senior* )  OR  ALL=( ageing )  OR  ALL=( aging )

)

AND

(

ALL=( “Companion robot*” )  OR  ALL=( “Assistive robot*” )  OR  ALL=( “Social robot*” )   OR  ALL=( “Virtual Reality” )  OR  ALL=( “Internet of Things” )  OR  ALL=( IoT )  OR  ALL=( Telemedicine )   OR  ALL=( Telemonitor* )  OR

ALL=( “Remote Technolog*”)  OR  ALL=( “Wearable Technolog*”)  OR   ALL=( “Sensing Technolog*”)  OR   ALL=( “Mobile Technolog*”)  OR  ALL=( “Information Technolog*”)  OR   ALL=( “Conversational Technolog*”)  OR   ALL=( “Gaming Technolog*”)  OR   ALL=( “Remote System*”)  OR   ALL=( “Wearable System*”)  OR   ALL=( “Sensing System*”)  OR   ALL=( “Mobile System*”)  OR   ALL=( “Information System*”)  OR   ALL=( “Conversational System*”)  OR   ALL=( “Gaming System*”)  OR   ALL=( “Remote Device*”)  OR   ALL=( “Wearable Device*”)  OR   ALL=( “Sensing Device*”)  OR   ALL=( “Mobile Device*”)  OR   ALL=( “Information Device*”)  OR   ALL=( “Conversational Device*”)  OR   ALL=( “Gaming Device*”)  OR   ALL=( “Remote Tool*”)  OR   ALL=( “Wearable Tool*”)  OR   ALL=( “Sensing Tool*”)  OR   ALL=( “Mobile Tool*”)  OR   ALL=( “Information Tool*”)  OR   ALL=( “Conversational Tool*”)  OR   ALL=( “Gaming Tool*”)  OR   ALL=( “Remote Application*”)  OR   ALL=( “Wearable Application*”)  OR   ALL=( “Sensing Application*”)  OR   ALL=( “Mobile Application*”)  OR   ALL=( “Information Application*”)  OR   ALL=( “Conversational Application*”)  OR   ALL=( “Gaming Application*”) OR   ALL=( “Fitness Tracker*”)

)

AND

(

ALL=( Well-being )  OR  ALL=( Wellbeing )  OR  ALL=( “Emotional health” )  OR  ALL=( “Mental health” )  OR  ALL=( Psychosocial )  OR  ALL=( Psycho-social )  OR  ALL=( “Limitation of Activit*” )  OR  ALL=( Self-efficacy )  OR  ALL=( Self-management )  OR  ALL=( Empowerment )  OR  ALL=( “Daily living” )  OR  ALL=( ADL )  OR  ALL=( “Community integration” )   OR  ALL=( “Social integration” )  OR  ALL=( “Social participation” )  OR  ALL=( Self-concept )   OR  ALL=( Self-esteem )  OR  ALL=( Self-perception)  OR  ALL=( Self-confidence )  OR  ALL=( “Psychological well-being” )  OR  ALL=( “Psychosocial Function*” )  OR  ALL=( Psychosocial )

)

AND

(

ALL=( “Clinical trial” )  OR  ALL=( “Multicenter study” )  OR  ALL=( “Controlled trial” )  OR  ALL=( “Randomized trial” )  OR  ALL=( “Clinical trial” )  OR  ALL=( “Comparative study” )  OR  ALL=( Random* )  OR  ALL=( Sham )      OR  ALL=( Controlled Before-After Studies)       OR  ALL=( evaluation study)    OR  ALL=( multicenter study)       OR  ALL=( case reports)       OR  ALL=( clinical study )

)

AND

(

DT=(Article)

)
