# Supplementary material for: Effect of Active and Assisted Living technologies on psychosocial well-being in older adults: systematic review
Source: Front Public Health. 2026 Jan 8;13:1717154. doi: 10.3389/fpubh.2025.1717154 (PMC12823859; doi:10.3389/fpubh.2025.1717154)
Supplement: Supplementary file 3 [file Table_2.docx]

**Supplementary Table 2 – Technological Components of AAL technologies**

| **Study** | **Input sources** | | | **Virtual Environment** | **Software Application** |
| --- | --- | --- | --- | --- | --- |
|  | **Wearable Sensing Device** | **non-Wearable Sensing Device** | **Manual imputation** |  |  |
| Benham S et al, 2019 (26) |  | Two hand controllers |  | Head-mounted display. Game playing controlled through hand controllers. | Computer Games (from commercial platforms - https:// [www.viveport.com](http://www.viveport.com) and <https://store.steampowered.com/> - tailored to participants’ occupational interests) for distraction from discomfort |
| Boer L et al, 2019 (36) |  | Micro-spirometer (*PiKo®-1*, nSpire Health, Inc., United States) | Pulse-oximeter (*CMS50D*, Contec Medical Systems CO Ltd, China). *Yes-or-No* questions concerning changes in symptoms, physical limitations, and emotions |  | Smartphone App (developed by the research team) for behaviours modification |
| Chan RSM et al, 2024 (32) |  | Smartphone-integrated front-facing camera (Commercially available, model not specified) |  |  | Mobile app (*e-Oral*, developed by the research team) for wellness (it helps users achieve the target motion properly during training) |
| Dieter V et al, 2024 (35) | Two Accelerometers (part of the *Re.flex* system, Re.flex, Austria) for motion tracking |  |  | Smartphone screen Avatar controlled via movements. | Smartphone App (*Re.flex*) for point of care |
| Ginis P et al, 2016 (40) | 9 axis IMU (*EXL-S3*, EXEL srl, Italy) for motion tracking |  |  |  | Two smartphone Apps (*ABF-gait* and *FOG-cue* - only for subjects with FOG, both developed by the research team) for behaviours modification |
| Gschwind Y et al, 2015 (27) | 3-axis accelerometer and barometer (*Senior Mobility Monitor*, Philips, the Netherlands) for motion tracking | Motion capture sensor (*Kinect*, Microsoft Corp, United States) |  | TV screen. Avatar controlled via movements, voice or tablet. Instructions and immediate performance feedback provided. | Computer Games for physical activity and Tablet App for education and behaviours modification (both specifically developed by the research team) |
| Keogh A et al, 2024 (38) | Smartwatch (*Charge 4*, Fitbit Inc, United States) measuring heart rate and activity | Smart weighing scale (*Aria Air*, Fitbit Inc, UNITED STATES) | Questionnaire for symptoms monitoring |  | Smartphone App (developed by the research team) for monitoring, wellness, behavior modification (mediated by medical intervention), compliance and education |
| Lee M et al, 2015 (34) |  | Motion capture sensor (*Kinect*, Microsoft Corp, UNITED STATES) |  | TV screen. Avatar controlled via movements. Visual and auditory feedback for movement correction. | Gaming console (*Xbox 360*, Microsoft Corp, UNITED STATES) game (*Zen Energy* exercise program from *Your Shape: Fitness Evolved*, Ubisoft Inc., Surrey, United Kingdom) for physical activity |
| Liljeroos M et al, 2024 (39) |  | Smart weighing scale (model not specified) | Questionnaire for symptoms monitoring (self-assessed levels of shortness of breath, fatigue, and oedema) |  | Tablet App (*OPTILOGG*, CareLigo AB, Sweden) for education and behaviours modification |
| Lim JS et al, 2023 (28) |  | Camera | User’s feedback through a tablet | Social robot with expressive LCD eyes, vocal interaction, and programmable movements within a narrative-based engagement |  |
| Park YH et al, 2021 (33) | Wristband (*NeoFit*, KT, Republic of Korea) for activity tracking | Blood Pressure monitor and Blood Glucose meter (commercially available, models not specified) | Diary for diet tracking |  | Smartphone App and a web-based platform (developed by the research team) for monitoring, wellness, behavior modification and education |
| Radhakrishnan K et al, 2021 (29) | Wristband (*Go*, Withings, France) for activity tracking | Smart weighing scale (*Body*, Withings, France) |  |  | Smartphone App (*Heart Health Mountain,* developed by the research team) for wellness (it is a sensor controlled digital game) |
| Seok JW et al, 2022 (30) | Smart watch with a 6-axis IMU (model not reported, DNX Co. Ltd, Republic of Korea) to detected movement, posture, and step-count | Touch sensors *(Touch tag, DNX Co*. Ltd, Republic of Korea) attached to key household items (e.g., refrigerator, microwave, sink, toilet, TV remote) |  |  | Smartphone App (developed by the research team) for monitoring, wellness and behavior modification. A version for care-receivers shows key indicators and highlights behavior anomalies, and is used to deliver voice messaging. |
| Wu R et al, 2024 (37) | Smartwatch (*Galaxy Watch*, Samsung, Republic of Korea) for measuring heart rate and activity  Smartphone (*Note 9*, Samsung) for measuring oxygen saturation |  | Diary for cough tracking |  | Smartphone App (developed by the research team) for monitoring, wellness, behavior modification and education |
| Yang WC et al, 2016 (31) |  | Wireless Balance board (model not specified) |  | Touchscreen monitor. Game playing controlled through center of pressure weight shift | Computer Game (origin not specified) for physical activity |

**Legend**

IMU Inertial measurement unit

FOG Freezing of gait
